# The Sticking Point in the Bench Press, the Squat, and the Deadlift: Similarities and Differences, and Their Significance for Research and Practice

**DOI:** 10.1007/s40279-016-0615-9

**Published:** 2016-09-06

**Authors:** Justin Kompf, Ognjen Arandjelović

**Affiliations:** 1 Kinesiology Department, Park Center, State University of New York at Cortland, Cortland, NY 13045 USA; 2 School of Computer Science, St Andrews University, St Andrews, Fife, KY16 9SX Scotland, UK

## Abstract

Since it was first observed, and especially so in recent years, the phenomenon of the so-called “sticking point” in resistance training has attracted a substantial amount of sports and exercise science research. Broadly speaking, the sticking point is understood as the position in the range of motion of a lift at which a disproportionately large increase in the difficulty associated with continuing the lift is experienced. Hence the sticking point is inherently the performance bottleneck, and is also associated with an increased chance of exercise form deterioration or breakdown. Understanding the aspects of lifting performance which should be analysed in order to pinpoint the cause of a specific sticking point and therefore devise an effective training strategy to overcome it is of pervasive importance to strength practitioners, and is conducive to injury avoidance and continued progress. In this paper, we survey a range of physiological and biomechanical mechanisms which contribute to the development of sticking points, and then, led by this insight, review and analyse the findings of the existing observational research on the occurrence of sticking points in three ubiquitous exercises: the bench press, the squat, and the deadlift. The findings of our analysis should be used to inform future research and current resistance training practice.

## Key Points

**Table:** 

A thorough understanding of the physiological and biomechanical mechanisms which contribute to the development of a sticking point is crucial in the analysis of athletic performance, and should guide the design of training strategies aimed at overcoming an observed performance bottleneck.
Contrary to what might be expected, currently available evidence suggests no substantial change in the electromyographic activity of muscles involved in a lift near the sticking point for all three exercises considered in the present article.
Although the location of the sticking point within the range of motion of a particular exercise varies significantly across different athletes, in the trained population, evidence suggests stratification by exercise execution style governed by personal biomechanics, with remarkable similarity in sticking point characteristics within each stratum.

## Introduction

The “sticking point” (or sometimes the “sticking region”; for a thorough discussion of the differences and their implications in the analysis of the phenomenon, see the work by Kompf and Arandjelović [6]) is a concept commonly used in the context of weight training [3–5]. Broadly speaking, it refers to the part of the range of motion (ROM) in a resistance exercise in which a disproportionately large increase in the difficulty associated with continuing the lift is experienced. More formally, in this work we adopt the sticking point definition proposed by Kompf and Arandjelović [6] as the point at which failure occurs when exercise is taken to the point of momentary muscular failure. Different forms of this definition were previously described by various authors such as Blackburn and Morrissey [7] and Cotterman et al. [8]. If the exercise is performed to exhaustion, given that failure is by the aforementioned definition experienced at the sticking point, two important practical concerns can immediately be observed. The first of these regards performance. If the sticking point is the proverbial weakest link in the execution of an exercise, it is the limiting factor which can have a profound effect on the load an athlete can employ in training or—in the case of athletes who compete in sports which inherently involve weight lifting (e.g. weightlifting and powerlifting)—which can directly impact competitive achievement. The second important concern is that of safety and injury prevention. A disproportionate increase in the difficulty of the lift, often coupled with a biomechanically weak ROM in which the sticking point occurs [9], increases the chance of exercise form breakdown and consequently injury. Therefore, understanding the multitude of factors which play a role in the development of sticking points [10, 11], as well as different strategies which a trainee can employ to remedy the associated weaknesses, are of major importance to strength training practitioners. In the present article we review the existing observational research on three exercises widely performed by different types of trainees: the bench press, the squat, and the deadlift. This review is used to highlight similarities and differences in the manner in which the sticking point in the three exercises is exhibited, and thus derive useful insight into the physiological and biomechanical factors of interest to resistance training researchers and practitioners.

## Preliminaries: Key Model Components

The phenomenon described by the term “sticking point” is underlain by complex interactions between different contributing factors which exhibit a high degree of exercise specificity. To explain a specific sticking point or to devise a training strategy to overcome it requires an understanding of these underlying factors as well as the biomechanics of the exercise in question. The present section builds the foundations of this understanding by explaining the key physiological and biomechanical mechanisms of significance in this context.

### Muscular Force

Muscles as functional units effect motion of the human body or its parts, including motion against resistance, by virtue of the contractile force they produce. A detailed review of the intermuscular architecture and the corresponding models of force generation is outside the scope of the present paper; for further detail we refer the interested reader to one of a number of recent reviews of the topic, e.g. those by Huxley [12], Cooke [13], and Piazzesi et al. [14]. Herein we constrain ourselves to a brief summary of the key elements.

The force produced by a given muscle is proportional to the number of sarcomeres in parallel within the muscle or, equivalently, the cross-sectional area (CSA) of the muscle (often referred to as the anatomical cross-sectional area or ACSA):

1 $$\begin{aligned} F_{\rm muscle} \propto A_{\rm CS} \quad \text { and }\quad A_{\rm CS}=\frac{V}{l}, \end{aligned}$$

where *V* is the volume of the muscle, *l* its length, and $A_{\rm CS}$ its ACSA.

Estimates of the maximal contractile force per unit of muscle range widely, from approximately 20 to 135 N/cm$^2$ [15–17], and the question of whether this potential maximum is the same across all skeletal muscles remains open. In contrast to muscles with a parallel myocyte (more commonly and henceforth called “muscle fibre”) architecture (strap and fusiform muscles), the fibres of which run in line with the force-generating axis of the muscular unit as a whole, fibres in muscles with a pennate structure insert into the tendon at an angle (pennation angle) which means that their effective force (true muscle force or tendon muscle force) is further modulated by the cosine of the pennation angle $\alpha _{\rm penn}$ [18]:

2 $$\begin{aligned} F_{\rm true} = F_{\rm muscle} \cos {\alpha _{\rm penn}}. \end{aligned}$$

When discussing pennate muscles, it is often more useful to adopt the use of the concept of the physiological cross-sectional area (PCSA), which is the area of a slice perpendicular to all of the fibres of the muscle (hence $A_{\rm CS} \le A_{\rm PCS}$).

Underlying the aforementioned universal mechanisms for force generation, the force produced by skeletal muscles is further affected by the following key factors:

- Force–length relationship
- Force–velocity relationship
- Fatigue
- Fibre recruitment
- Fibre type

We explain each of these in turn next.

#### Force–Length Relationship

**Fig. 1:**
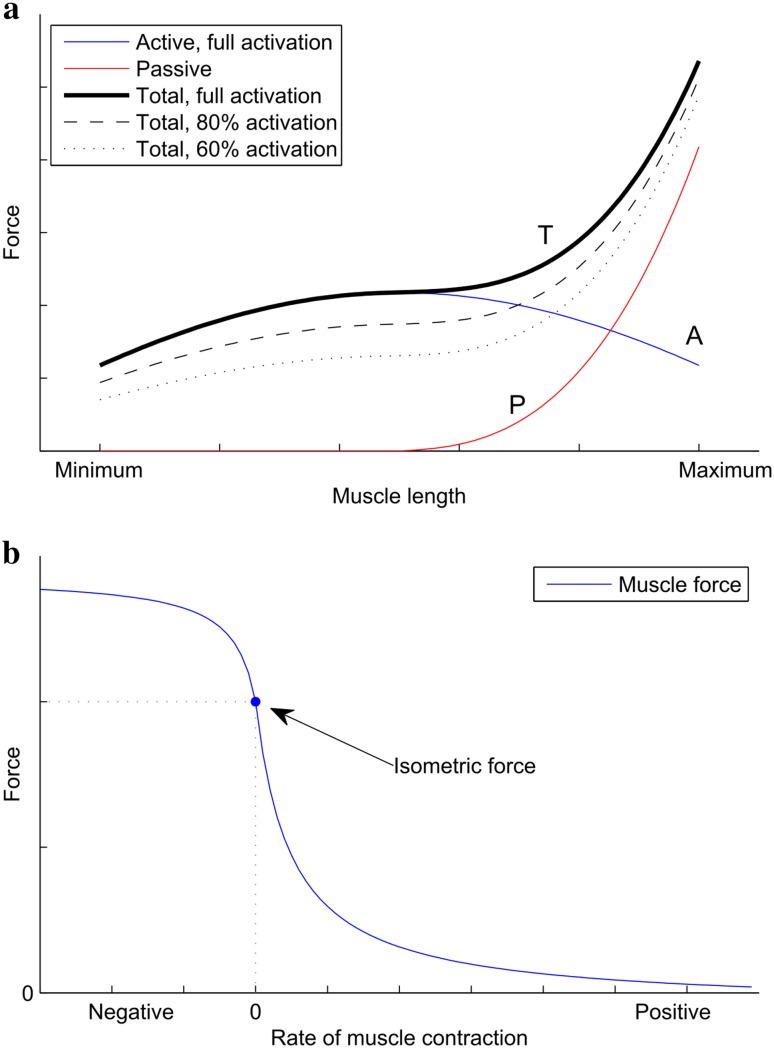
**a** A typical force-length diagram (not to scale) for an isolated striated muscle [1]. Two components contributing to total force production (T, *black*) are shown: active (A, *blue*) and passive (P, *red*). Total forces for different levels of muscle activation are shown in* black* in different styles (100 %—*solid*, 80 %—*dashed*, 60 %—*dotted*). **b** A typical force–velocity diagram (not to scale) for an isolated striated muscle [2]

It is well established that the maximal force that an individual skeletal muscle can produce varies with the extent of its elongation [19, 20]. Both shortening or stretching the muscle from its optimal length for force production (usually its relaxed length) tends to reduce the active force from its maximum by, respectively, excessive or insufficient actin and myosin filament overlap [21]. In contrast, the passive component of muscular force increases with elongation, resulting in a typical overall force–length characteristic illustrated in Fig. 1a. This means that the force a lifter can apply against the bar varies throughout the lift in a manner independent of leverage changes due to the biomechanics of the lift.

#### Force–Velocity Relationship

In addition to its dependence on the instantaneous length of a muscle, muscular force is also dependent on the rate of change of muscle length, i.e. the contraction velocity [2, 22]. Specifically, the ability of skeletal muscle to produce force decreases as the velocity of contraction increases. Maximum force is produced for rapid eccentric contractions, it is reduced for isometric contraction, and yet further (approximately hyperbolically) for concentric contractions. The typical force–velocity characteristic for an isolated muscle is shown in Fig. 1b.

#### Fatigue

Finally, as it produces force, a muscle experiences fatigue—a decrease in maximal force and power that it can produce. This decrease begins shortly after the onset of contractions [23], and its rate over time is dependent on the magnitude of force exerted and its duration [24, 25]. Fatigue is governed by a complex multifactorial process which involves both central and peripheral factors [26] affected by changes to the central nervous system drive to motor neurons, the muscles and motor units in use, neuromuscular propagation, excitation contraction coupling, intramuscular milieu, muscle blood flow, and substrate (e.g. creatine phosphate and carbohydrate) availability [23]. For further discussion of the effects of fatigue on the location of the sticking point, the reader is referred to [35, 37].

#### Fibre Recruitment

The factors which influence muscular force we have described so far can be characterised as pertaining to the low-level biophysical architecture of the muscle. No less important are neural factors. The force of contraction of a muscle as a whole is dictated by (i) the frequency of stimulation (rate coding) coming from the motor nerves (motoneurons) which innervate muscle fibres [27], and (ii) by the number of active motor units [28]. A single low-intensity stimulus effects a twitch contraction of a small number of smaller motor units [29]. If the stimulus is repeated before the muscle relaxes, a sustained contraction occurs; this is referred to as tetanic contraction. With the increase in the intensity of the stimulus, the number of stimulated motor units is also increased, and progressively larger motor units are recruited [29]. In most cases, at 85 % of maximal voluntary force, nearly all motor units are recruited [30], although this proportion may be much lower for some muscles [27, 28]. When a submaximal voluntary force is sustained, and as fatigue accumulates, motor unit recruitment increases to maintain force output [31, 32].

#### Fibre Type

Finally, the ability of a muscle to produce and sustain force is dependent on the type of fibres it comprises. While it should be noted that there are numerous ways in which muscle fibres can be categorised (e.g. based on their metabolic properties, phenotypical characteristics, histochemical or immunohistochemical staining responses, etc.), for the purpose of the present discussion we are referring to the most common categorisation into two broad groups, type I and type II fibres [33, 34], which exhibit different contractile properties. Generally, the innervating axon diameter and the magnitude of the maximal contractile force are smaller for type I fibres and larger for type II; so, as we noted in the previous section, according to Henneman’s size principle, the slower contracting but less rapidly fatiguing fibres of type I are recruited first, with faster twitch fibres progressively recruited as the resistance increases [29]. Note that nearly all muscle fibres in a motor unit are of the same fibre type.

### Torque

The manner in which forces produced by individual muscles allow an athlete to exert effective force against resistance is governed by the biomechanical context of the human body and the specific exercise, and inevitably involves force transfer by virtue of torque (sometimes also referred to as moment of force). Torque can be seen as a rotational analogue of force, and just as the force experienced by an object is defined as the rate of change of the object’s linear momentum [35], the net torque experienced by an object (e.g. a limb) is defined as the rate of change of the object’s angular momentum. In the context of the present work, the torque $\tau$ produced by a muscle around a point of interest (usually a joint) can be understood as being given by

3 $$\begin{aligned} \tau = r \times F, \end{aligned}$$

where, as usual, $\times$ denotes a vector cross-product, *F* is the muscular force, and *r* is the distance of the point of interest (centre of rotation) from the point at which the force is applied. This is sometimes referred to as internal torque. A similar relationship can be written between the external resistive force (e.g. the weight of a barbell) and the corresponding so-called external torque. Observe that, like force, torque is a vector. It is perpendicular to the plane defined by *r* and *F*, and its magnitude is given by

4 $$\begin{aligned} |\tau | = |r| |F| \cos {\phi }, \end{aligned}$$

where $\phi$ is the angle between the vectors *r* and *F*. Note that in the context of the present discussion, in most cases, our interest is in the torque around a joint effected by a muscle spanning that joint, in which case |*r*| is approximately constant, |*F*| is dictated by factors such as those reviewed in Sect. 2.1, and $\cos {\phi }$ changes in accordance with the constraints of an exercise and an individual’s biomechanics [36].

By examining the terms on the right-hand side in Eq. 3, it can be readily seen that muscular torque can be affected by changing either (i) the force that the muscle produces, (ii) the angle between the direction of force and the distance from the point of interest (chosen centre of rotation) to the point at which the force is applied, and (iii) the point at which the force is applied. As discussed in detail by Kompf and Arandjelović [6], this insight is crucial in the analysis of sticking points and in the design of effective training strategies which may be used to overcome them.

**Fig. 2:**
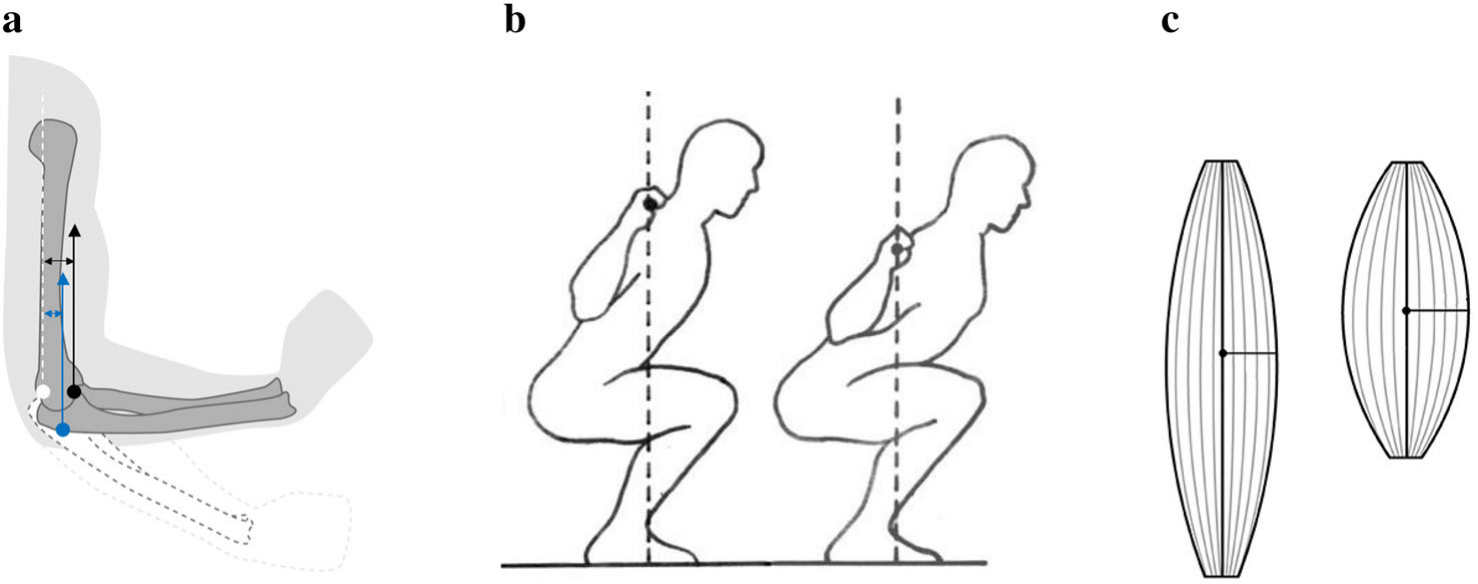
The relationship between force and the torque it effects is an important consideration at various structural and functional levels. **a** Changing effective levers and lines of pull affect both the torque effected by the external load and a muscle treated as a single force-producing unit [35]. **b** Alterations of exercise form, involving changes in the placement of the external load or the body positioning (e.g. stance or grip width), affect the amount of resistance experienced by different functional muscle groups. **c** Leverage and line of pull changes are also relevant on scales smaller than a muscle. Relative contributions of individual fibres vary through the range of motion of an exercise and are affected by hypertrophy

Although the concept of torque is pervasive in sports science, it is important to observe complexities which arise due to biomechanical changes that affect torque at different functional levels. Indeed, the analysis of even a simple single joint exercise such as the arm curl reveals qualitatively different patterns in demands placed on the muscles involved when the strength characteristics of a particular trainee and changes in the speed of the movement are taken into account [37]. Probably the most widely discussed biomechanically effected changes of torque are those which happen during the execution of an exercise by means of changes in effective levers or lines of action of both muscular and external forces, as illustrated in Fig. 2a. Indeed, in the context of the sticking point, in our previous research [6] we argued that, while important, these biomechanical factors alone fail to explain significant aspects of the collected observational data. Another way in which torque as a means of muscular force transmission across a joint can be changed pertains to the manner in which an exercise is performed. Altering the placement of the load (e.g. high vs. low bar squat) or the positioning of the body (e.g. conventional vs. sumo style deadlift) also alters effective levers or lines of action of different forces, thus placing different demands on contributing muscle groups, as illustrated in Fig. 2b. Lastly, much less discussed are line of pull changes associated with individual fibres within a muscle. As shown in Fig. 2c, as a muscle contracts (or indeed relaxes), different fibres’ lines of pull change by different amounts, thus affecting their relative contributions to the overall force exerted by the muscle. Such changes can be seen to be intricately tied to previously discussed changes associated with the overall biomechanics of the exercise, thereby demanding nuanced analysis in the context of a particular trainee and exercise of interest.

## Observational Research

As we noted in the previous section, the aetiology of a specific sticking point exhibits a high degree of exercise specificity. Despite this observation, the present research on sticking points is rather limited in its scope of consideration of different exercises. Nevertheless, it is insightful to start by reviewing observational research on the sticking point in the three exercises which have attracted a significant amount of research: the bench press, the squat, and the deadlift. This helps place the problem under consideration in the context of actual resistance training practice, identify some of the key challenges which need to be addressed, and illustrate the importance of sticking points in general.

### Bench Press

The bench press is one of the most popular exercises used to strengthen the musculature of the upper body, primarily the chest, shoulders, and arms. It is widely employed for general strength and conditioning, as well as hypertrophy. The bench press is performed with the athlete in a supine position, supported by a flat, horizontal bench. Traditionally, a straight free bar is used to impose resistance, although different means are also possible using a variety of machines [38].

The principal actions taking place during the concentric phase of the bench press are those of humeral adduction in the transverse plane, elbow extension, and scapulothoracic abduction, the extent of each exhibiting significant variation depending on the lifter’s biomechanics and exercise execution style [39]. Humeral adduction is primarily effected by the action of the pectoralis major, with a significant additional contribution from the anterior deltoid, and minor contributions from the biceps brachii and coracobrachialis. In addition, it is seldom noted that although it is unable to contribute to humeral adduction directly, the triceps brachii does make this contribution in the bench press by virtue of the kinematic constraints imposed by the closed-chain nature of the lift and the extensor action at the elbow.

#### Key Findings

Given the versatility and popularity of the bench press, it is unsurprising that the analysis of its biomechanics has attracted significant research attention. Indeed, to the best of the authors’ knowledge, the bench press in its various forms (using a barbell, dumbbell, or different types of resistance machines) is the only upper body exercise in which the sticking point has been studied [11].

Some of the earliest detailed analyses of the sticking point in the bench press were conducted by Wilson et al. [40] and Elliott et al. [9]. Using a robust data acquisition setup and a sample of elite male powerlifters, they found that for near-maximal loads the sticking point was exhibited near the beginning of the lift, on average at approximately 30 % of the bar height for a completed lift. Their findings also provide a good example of the potential safety concerns associated with the sticking point which we noted in Sect. 1. In particular, Wilson et al. observed a significant increase in the horizontal bar movement which occurred in the proximity of the sticking point, which indicates a potential technique breakdown. Despite the small sample size of 10 lifters used in this study, and while the location of the sticking point was very consistent across the majority of the lifters, the authors also observed that a number of lifters did not exhibit a sticking point, even at maximal effort. For these individuals, the lift was in a sense uniformly challenging through the entire ROM, suggesting that they could experience failure to complete the lift at any stage in the ROM.

Subsequent work by Wagner et al. [39] provides some insight into the apparent stratification observed by Wilson et al. [40]. Specifically, Wagner et al. examined the influence of the grip width on the performance and different biomechanical aspects of the bench press. They found that for the middle grip width, the sticking point was found to occur at a greater vertical distance from the shoulder axis and lasted for a smaller percentage of the ascent phase (11.4 %) than for either the narrow (17.3 %) or wide (22.5 %) grip widths. The same phenomenon has recently been reported by Gomo [41].

In contrast to the early-phase sticking point observed by Wilson et al. in elite powerlifters, in a study which involved trained recreational lifters, Król et al. [42] reported the occurrence of the sticking points closer to the midpoint of the ascent phase. On the surface this finding appears to contrast with that of Wagner et al. [39], particularly considering that the subjects in the study by Król et al. were asked to use the uniform grip with the 81-cm spacing between palms—the widest grip allowed by the International Powerlifting Federation (IPF). As we noted in the previous paragraph, wide grip in elite powerlifters was associated with a lower sticking point location. However, a closer look at the design of the two studies offers a potential explanation which resolves the apparent conflict of the findings. In particular, in the study by Wagner et al., the participants used their preferred grip, while (as noted before) the grip width was fixed by the authors in the study of Król et al. It is reasonable to expect that trained powerlifters, especially at an elite level, would have converged towards the grip that suits their body structure the best. In addition, for those participants in the study by Król et al. [42] who were not used to the assigned grip, in addition to any inherent unsuitability of their body structure, it is likely that the weaknesses associated with a nonpreferred exercise execution style would have been further amplified by the lifters not being accustomed to it.

Electromyographic muscle activity (EMG) and the activity pattern changes surrounding the sticking point region in the bench press have been studied by a number of researchers [9, 10, 43]. The findings are consistent. In particular, the pectoralis major, usually the most significant contributor to the bench press and the key muscle targeted by the exercise, exhibits strong activity throughout the concentric portion of the lift [3, 9]—usually with a slow but steady decrease with time [9, 11] which continues throughout the neighbourhood of the sticking point, sometimes with a short lasting spurt of increase at the sticking point itself [9]. The anterior deltoid, a major contributor to the lift as well, also shows strong activity throughout, with a significant increase around and following the sticking point [10, 42]. A similar increase in the activity of the triceps brachii has been reported by a number of authors [11, 42], though the finding has not been observed universally [3]. Observations regarding the activity of the biceps brachii appear inconclusive. Elliott et al. observed an increase and the peak in activity of the biceps brachii around the sticking point [9], while more recently van den Tillaar and Saeterbakken [11] reported the converse. A possible reason for this discrepancy lies in the design differences between the two studies. As noted in Sect. 3.1.1, the work by Elliott et al. used elite powerlifters. In contrast, the work by van den Tillaar and Saeterbakken employed students, none of whom had experience in competitive powerlifting, and some of whom had as little as one year of bench press training experience ($4.6\pm 2.2$ years for the entire cohort). Considering the generally inferior position of the bar at the beginning of the concentric phase of the bench press observed in the exercise technique practiced by powerlifters in comparison with the recreational training population, it is reasonable to expect a more significant activation of the biceps brachii (with this style in a more advantageous position to contribute to flexion around the shoulder joint).

### Squat

Owing to its biomechanical and neuromuscular similarities to a wide range of athletic and everyday tasks, the squat is one of the most widely used resistance exercises. It is frequently used for general strength and conditioning preparation in a variety of sports as well as in rehabilitation, and with the greatest specificity in the competitive sports of weightlifting and powerlifting. Notwithstanding significant biomechanical differences between different execution styles (based on the width of the stance [44], bar placement [45], and the orientation of the knee flexion planes [46], to name just a few), all variants of the squat involve synergistic hip and knee flexion in the descent to the desired depth, followed by knee and hip extension in the ascent which terminates with the lifter in the starting position [47, 48]. This makes the squat an exercise primarily aimed at training the muscles of the lower body, specifically the quadriceps femoris, rectus femoris, hip extensors, adductors, and abductors [49], though many more muscles are involved in various supporting roles such as stabilisation and balance [50].

#### Key Findings

The squat is a notoriously complex exercise both in terms of its biomechanics and its neuromuscular demands. Given that the resisted portion of the movement, the ascent, involves concentric actions of muscle groups with antagonist functions (e.g. the quadriceps and the hamstrings at the knee and the hip) [51], perfecting the squat requires much practice which fine-tunes the timing and the extent to which different contributing muscles are engaged. Considering this complexity, it is of no surprise that a sticking point has been repeatedly observed in the squat in numerous studies. Indeed, in the academic literature, the phenomenon of the sticking point was first reported and studied in the squat [52]. In particular, McLaughlin et al. [52] examined kinematic characteristics of the squat performed by highly skilled powerlifters, and observed that the sticking point across the studied sample occurred at approximately a thigh angle relative to the ground of $30^\circ$. What is more, they observed a remarkable uniformity across the sample in this regard, as witnessed by the standard deviation of the angle of only $\pm 2^\circ$. A virtually identical finding was recently reported by Hales et al. [53], who observed the sticking point at a thigh angle of $32^\circ \pm 2.0^\circ$ in a sample of competitive powerlifters of varying skill levels. In this study, the authors also measured the positions of other body segments. Of particular importance in the context of the present paper were the findings that both the trunk and shank angles relative to the ground exhibited much greater variation between different lifters, of $\pm 6.3^\circ$ and $\pm 7.3^\circ$ respectively, which highlights the interaction between the squatting style adopted by an athlete, the athlete’s biomechanics, and the point in the lift at which the athlete is most likely to exhibit a sticking point. This interaction was examined in depth by Escamilla et al. [44], who also performed a stratification of the studied sample into three groups by their stance width (normalised by shoulder width): narrow, medium, and wide. The first interesting finding of this work is the much greater thigh angle at the sticking point than that reported by McLaughlin et al. [52] and Hales et al. [53]: approximately $49^\circ$. Also contrasting the findings summarised before, Escamilla et al. observed a significantly greater variability of the sticking point thigh angle across lifters of $\pm (5^\circ$–$6)^\circ$. Particularly surprising was that much greater variation was found within the three groups (narrow, medium, and wide) than across groups (approximately $\pm 2^\circ$). Considering that the parameters of the studies by Escamilla et al. and McLaughlin et al. were very similar (they both used highly skilled powerlifters who wore one-piece squatting suits), an insight which would explain this discrepancy has yet to emerge; indeed, Escamilla et al. did not discuss this aspect of their findings.

Interestingly, in comparison with the bench press, studies of electromyographic muscle activity in the squat in the context of the sticking point are lacking. One of the few studies in this realm is that by van den Tillar et al. [54]. In contrast to the portion of the lift surrounding the sticking point in the bench press wherein significant changes (decreases for some and increases for others, see Sect. 3.1) to EMG muscle activity were observed, no similar trends were noticed in the squat. The rectus femoris and vastus lateralis, both significantly active in the squat, exhibited a steady decrease in activity throughout the lift (as previously reported by Escamilla et al. [55] and McCow and Melrose [56]), while biceps femoris, a lesser contributor, showed a slight and steady increase. The only muscle which did show some (albeit slight) change at the sticking point was the vastus medialis, the most activated muscle in the lift, which demonstrated a transient increase in activity. However, the authors’ statistical analysis as well as previous findings recorded in the literature [55, 56] suggest that this was a chance occurrence rather than a genuine pattern.

### Deadlift

Much like the two exercises discussed in Sects. 3.1 and 3.2, the bench press and the squat, the deadlift is a competitive lift in the sport of powerlifting. In addition to powerlifters, the deadlift is often used by weightlifters as an assistance exercise [57], by bodybuilders to stimulate the hypertrophy of the muscles of the back and the thighs [58], as well as by a range of athletes for the development of general strength [59].

Much like in the resisted phase of the squat, the primary dynamic actions taking place in the deadlift are hip and knee extension. Hip extension is primarily effected by the gluteus maximus, and the biceps femoris (the long head), semitendinosus, and semimembranosus muscles, whereas knee extension is achieved by the action of the vastus medialis, vastus lateralis, vastus intermedius, and rectus femoris. Concurrently with hip and knee extension, there is a significant static engagement of the interspinales and multifidus, and the erector spinae complex, i.e. the iliocostalis lumborum, longissimus dorsi, iliocostalis thoracis, and spinalis dorsi. Numerous other muscles are engaged as lesser contributors or stabilisers, such as the rotatores, intertransversarii, biceps brachii, latissimus dorsi, etc. Notwithstanding the aforestated apparent similarity with those of the squat, the biomechanics of the deadlift exhibit significant differences. In particular, the maximal knee flexion angle tends to be smaller in the deadlift, which places a greater emphasis on hip flexion to complete the lift. In addition, the ascent in the deadlift is not preceded by a descent—the weight starts at rest (“dead” weight) on the floor. Lastly, unlike the bench press and the squat, the deadlift is seldom performed using anything other than a free weight (usually a barbell).

Though the difference may in part be a normative one, it is generally recognised that the style in which the deadlift is performed exhibits less variation than for the squat [60], the two most prominent being the so-called conventional style and the sumo style [60]. The former is characterised by a narrower stance (approximately shoulder width) and the feet in 10–15° of external rotation [47], and the latter by a 2–3 times wider stance and the feet in 40–45° of external rotation [47].

#### Key Findings

As regards the phenomenon of the sticking point, of the three lifts discussed herein, the deadlift has so far received the least amount of attention. Nevertheless, the published studies on the topic do provide interesting findings, some of which exhibit a degree of universality and thus similarity with the findings in the previous two sections, and some of which highlight a number of characteristics specific to the deadlift. In addition to the squat, in the work already mentioned in the previous section, Hales et al. [53] also examined the kinematics of the deadlift performed by the same sample of competitive powerlifters of varying skill levels. The observed thigh angle of $60^\circ$ (relative to the ground) at the sticking point was much greater than in the squat (approximately $30^\circ$), again with a remarkable consistency across different lifters, as witnessed by the small standard deviation of $3^\circ$ across the sample. Much like in the squat, the trunk and shank angles at the sticking point exhibited greater variability (standard deviation of approximately $7^\circ$) but with a much greater mean (approximately $60^\circ$ vs. $40^\circ$ for the deadlift and the squat respectively) for the trunk and a somewhat lower mean for the shank (approximately $75^\circ$ vs. $70^\circ$ for the deadlift and the squat respectively). These observations illustrate the already noted emphasis on hip extension in the deadlift, and suggest that this feature of the movement may also be key to addressing its sticking point. A particularly insightful observation made by Hales et al. is that while in the squat the knee and hip flexion angles exhibited a highly correlated linear change throughout the movement, the kinematics of the conventional deadlift were more segmented, showing three phases in the lift. The first phase is dominated by knee extension (a significant change in the knee flexion angle and a small change in the hip flexion angle), the second one by hip extension (a significant change in the hip flexion angle and a small change in the knee flexion angle), and in the third and final phase, the knees and the hip extend in unison. This observation simplifies the analysis of the sticking point in the deadlift and can be of major value in identifying the appropriate strategy to overcome it [6].

The recent work by Beckham et al. [61] offers interesting insight into the phenomenon of the sticking point in general as well as specifically in the context of the deadlift. In this study, the authors assessed the isometric strength in key positions in the deadlift in a sample of competitive powerlifters. They found that the starting position was the weakest of all, with an average peak force of approximately 3400 N, followed by the position for which the bar is at the knee level (an average peak force of approximately 4100 N) and the lockout (an average peak force of approximately 4900 N). The strongest position was found to be at the point at which the bar is at the mid-thigh level (an average peak force of approximately 5800 N, or 70 % greater than at the weakest, the starting position). The same trend was observed even after allometric scaling by the body mass of the lifter was applied. When these findings are compared with kinematic analyses of the actual lift (e.g. by McGuigan and Wilson [62]), several observations can be made. Firstly, they illustrate that the sticking point does not occur at the weakest position, even if that position is at the very beginning of the lift. This reinforces the argument made [6] pertaining to the manner in which the sticking point is defined and understood in the literature. Secondly, they highlight the importance of what can be described as lifting context, i.e. the interaction between different biomechanical and physiological factors which affect the ability of a muscle to produce force, and the complexity of trying to understand, explain, and indeed overcome a specific sticking point [6].

## Summary and Conclusions

In this paper we have focused our attention on the phenomenon of “sticking points” observed in resistance training. Owing to their performance-limiting aspect in competition and training, as well as their significance in the context of injury prevention and rehabilitation, sticking points have attracted a great deal of attention both in the academic community and in practice. The presented consolidation of sticking point related research in the context of three major exercises used by a diverse training population should aid researchers in guiding future work, and instruct and inform strength practitioners using the most comprehensive body of evidence surveyed thus far.

Our article makes several important contributions. We started by reviewing the key physiological and biomechanical mechanisms which can contribute to the development of a sticking point. A thorough understanding of the aforementioned mechanisms is crucial in the analysis of athletic performance and should guide the design of training strategies aimed at overcoming an observed performance bottleneck. Informed by this insight, we reviewed and consolidated the existing body of observational work on the three popular exercises which dominate sticking point research: the bench press, the squat, and the deadlift.

Our consolidation of the existing literature revealed a number of interesting findings and highlighted promising areas for future research. The first insight we wish to highlight concerns the location of the sticking point in a particular exercise across different athletes. We found that while there appears to be significant variability in this regard, trained individuals tend to converge towards exercise execution styles which best fit their biomechanics, resulting in stratification of performance characteristics, with remarkable uniformity of lifting characteristics within each stratum. This observation has several practical implications. For example, the within-stratum uniformity simplifies the analysis of the sticking point and the associated remedial exercise prescription.

Another possibly surprising finding concerns EMG patterns. Though imbalanced across different exercises considered here, the available evidence suggests that there are no significant changes around the sticking point in the EMG activity of main muscles contributing to the movement. That being said, there is a noteworthy absence of studies specifically examining EMG patterns in the context of the sticking point for the deadlift—by far the greatest amount of work has focused on the bench press. Future work should seek to address this gap in observational evidence.
